# Mpox vaccination in global perspective: priorities and challenges

**DOI:** 10.1097/MS9.0000000000000550

**Published:** 2023-04-01

**Authors:** Jigyasa Rana, Shailesh Kumar Patel, Aditya Agrawal, Nikhil K. Channabasappa, Ankush K. Niranjan, Bhabesh Chandra Das, Megha K. Pandey, Sita Prasad Tiwari, Milan Gaihre

**Affiliations:** a Department of Veterinary Anatomy, Faculty of Veterinary and Animal Sciences, RGSC, Banaras Hindu University, Barkachha, Mirzapur, Uttar Pradesh; Departments of b Veterinary Pathology; c Veterinary Physiology and Biochemistry; d Veterinary Microbiology; e Veterinary and Animal Husbandry Extension, College of Veterinary Science and Animal Husbandry, Rewa; f Department of Translational Medicine, All India Institute of Medical Sciences, Bhopal; g Nanaji Deshmukh Veterinary Science University, Jabalpur, Madhya Pradesh, India; h Department of Internal Medicine, College of Medical Sciences, Kathmandu University, Chitwan, Nepal

**Keywords:** containment, global approach, monkeypox, vaccination

## Abstract

After the global panic created by COVID-19, the monkeypox (Mpox) virus emerged as a new challenge for the world population. As of 19 January 2023, a total of 84,733 cases across 110 countries/territories including 80 deaths has been reported. The virus has been transmitted to nonendemic countries in a short span of 6 months warranting WHO to declare Mpox, a Public Health Emergency of International Concern on 23 July 2022. As the Mpox virus is crossing geographical boundaries without established transmission patterns, there is an urgent need for new scientific strategies from global researchers to contain it before turning into the next pandemic. The control of Mpox outbreaks primarily relies on various public health measures such as proper surveillance, contact tracing, rapid diagnosis, isolation and care of patients, and vaccination. At present, there are three vaccines viz. ACAM2000, MVABN, and LC16 are in consideration and have been approved in several jurisdictions for ongoing Mpox outbreak. Prioritization of individuals along with the production of specific Mpox vaccine is need of the hour to meet out the global demand of Mpox vaccination.

After the global panic created by COVID-19, the monkeypox (Mpox) virus emerged as a new challenge for the world population. As of 19 January 2023, a total of 84 733 cases across 110 countries/territories including 80 deaths has been reported^1^. The virus has been transmitted to nonendemic countries in a short span of 6 months warranting WHO to declare Mpox, a Public Health Emergency of International Concern on 23 July 2022. Surprisingly, the recent outbreaks do not follow the established pattern of spread in which the Mpox virus was distributed to West and Central Africa near tropical jungles by immigration of people from Africa or exposure to infected exotic pets^2^. As the Mpox virus is behaving differently, crossing geographical boundaries without established transmission patterns, there is an urgent need for new scientific strategies from global researchers to contain it before turning into the next pandemic.

The Mpox virus disproportionately affected marginalized communities and at present global risk of the disease is assessed as moderate by the WHO. However, the European and American regions are at moderate and higher risk of the disease respectively, the Asian and African countries also need to prioritize preparedness for the virus. The Mpox virus is a brick-shaped, enveloped, double-stranded DNA virus, belongs to the genus *Orthopoxvirus* of the *Poxviridae* family, related to the vaccinia, variola, and cowpox virus. The genome of the Mpox virus consists of 197.209 kbp comprising of identical but inverted 6379 bp terminal repetition in the genome, coding for genes involved in host range determination and pathogenesis along with conserved central regions coding for replication and assembly machinery^3^. It has more than 185 open reading frames translated to 60 amino acid residues^4^. Formerly, based on their virulence and different topography, the viral strains divided into two different genetic clades: Central African (Congo Basin) clade viz. Clade I and West African clade viz. Clade II. Clade I strain caused more severe disease and is more transmissible than Clade II. The only country where both virus clades found was Cameroon. Recently, Clade III which is a subdivision within the former Clade II was reported during 2017–2019 from different outbreaks in Nigeria, USA, Singapore, UK, and Israel^5^. The ongoing Mpox outbreak has been associated with Clade II of the Mpox virus, which is less virulent than Clade I^6^.

The Centers for Disease Control and Prevention (CDC) pointed out the risk of airborne infection of the Mpox virus through respiratory droplets along with direct contact and contaminated environments like bedding, towels, clothing, objects, electronics, and surfaces^7^. The spread of infection through close contact with someone who is infected with Mpox, including kissing, intimate or sexual contact is also a matter of serious concern, as the virus has also been reported in semen^8^. Surprisingly, most cases of Mpox are men who have sex with men (MSM) in connected social and sexual networks. Notably, the individual remain infectious until all their lesions become crusted and scabs have fallen off, after the formation of a new layer of skin underneath, giving the prolonged window to the virus for further spread.

The control of Mpox outbreaks primarily relies on various public health measures such as proper surveillance, contact tracing, rapid diagnosis, isolation and care of patients, and vaccination. At present, there are three vaccines are in consideration and have been approved in several jurisdictions for ongoing Mpox outbreak. All the three vaccines viz. ACAM2000, MVA-BN (also known as Imvamune, JYNNEOS, or Imvanex), and LC16 were developed against smallpox, and their cross-protection against Mpox needs to be evaluated. Among the three vaccines used against Mpox, MVA-BN is a nonreplicating vaccine administered as two doses of subcutaneous injection of 0.5 ml each given at least 4 weeks apart, whereas LC16 and ACAM2000 are minimally replicating vaccines, and replicating vaccinia-based vaccines, respectively administered as a single dose using the scarification method. Keeping the risk assessment by WHO in consideration, mass vaccination is not recommended at the present scenario but primary preventive vaccination is recommended for the high-risk group such as gay, MSM, individuals with multiple casual sexual partners, sex workers, healthcare personnel involved in diagnosis and treatment, and members of the outbreak response team. In addition, children, pregnant women, and immunocompromised individuals should be prioritized for primary preventive vaccination only when they are at high risk of exposure. The postexposure preventive vaccination is recommended for contacts of confirmed cases ideally within the first 4 days of exposure up to 14 days in the absence of symptoms. Children, pregnant women, and immunocompromised individuals should be offered postexposure preventive vaccination in a priority as they are at risk of developing more severe Mpox disease when infected.

For healthy individuals, all the three vaccines are appropriate but in individuals with severe immune deficiency, only MVA-BN should be used. Immunocompromised individuals including those with cancers, transplants, immunodeficiency diseases, and on immunosuppressive medication are at high risk of infection and should be prioritized for MVA-BN vaccination. In pregnant and breastfeeding women, only MVA-BN should be used for primary and postexposure preventive vaccination. However, MVA-BN and LC16 can be used in children for postexposure vaccination as it is authorized by USA and Japan respectively but children should only be vaccinated when the benefits of vaccination significantly outweigh the potential risks. Older individuals should be vaccinated irrespective of their history of smallpox vaccination if exposed or at higher risk.

As the cases of Mpox are continuously increasing, the supply constraint of vaccines in some countries owing to several policy and regulatory issues such as safety studies, clinical trials, common side effects, vaccine schedules, vaccine package inserts, authorization procedures, etc. are a major concern for the global community. In case of vaccine shortage, close contacts of Mpox, children, pregnant women, and immunocompromised persons should be prioritized for vaccination after a proper analysis of risks and benefits. Dose-sparing options such as immunization by intradermal route to increase the number of doses by five-fold may prove crucial in the control of the disease. Individuals with two doses of preexposure vaccination need not to receive postexposure vaccination to spare it for other high-risk individuals. Ring vaccination may prove crucial if infectious cases are promptly diagnosed and the individuals are vaccinated within 1–4 days of exposure.

The major challenges in Mpox vaccination include unknown contacts of cases and tracing contacts within a recommended period (2 weeks) for postexposure immunization. The limited availability of the available vaccines is another challenge which can be addressed by prioritization of vaccination for individuals who are at greater risk of the disease. Although the Mpox virus poses the highest risk to sex workers, especially MSM, they have not been prioritized in vaccination campaigns. Looking into the disproportionate burden of infection on sex workers across diverse social settings there is an urgent need to prioritize them for Mpox vaccination. The issues related to safety, immunogenicity, efficacy, and dose-sparing options need to be addressed on priority. The collaborative global effort in the direction to develop more vaccines against the Mpox is need of the hour. In this direction, a total of 11 clinical trials are currently evaluating the efficacy and safety of Mpox vaccines (Table 1). Moreover, an increased number of clinical trials and identification of new vaccine candidates against the virus is of utmost necessity to fulfill the global vaccine demand. The biological and epidemiological understanding of the Mpox virus in the context of different countries should be strengthened for the determination of their clinical and public health needs regarding vaccine production, along with operational requirements.

**Table 1:** Clinical trials evaluating the efficacy and safety of Mpox vaccines (https://www.clinicaltrials.gov)

S. no.	NCT number	Title	Phase	Interventions	Populations	Location
1	NCT02977715	IMVAMUNE® smallpox vaccine in adult healthcare personnel at risk for monkeypox in the Democratic Republic of the Congo	Phase 3	Biological: IMVAMUNE	Enrollment: 1600 Age: 18 years and older (adult, older adult) Sex: All	Tshuapa site, Boende, Tshuapa, The Democratic Republic of the Congo
2	NCT05438953	Follow-up of contact at risk of monkeypox infection: a prospective cohort study	NA	Biological: vaccination with MVA vaccine (IMVANEX® and JYNNEOS®)	Enrollment: 226 Age: 18 years and older (adult, older adult) Sex: All	CIC Cochin-Pasteur, Paris, France
3	NCT05512949	Trial to evaluate the immunogenicity of dose reduction strategies of the MVA-BN monkeypox vaccine	Phase 2	Biological: JYNNEOS	Enrollment: 210 Age: 18–50 years (adult) Sex: All	University of California, San Diego – Antiviral Research Center, San Diego, California, USA The George Washington University Medical Faculty Associates – Infectious Diseases, Washington, District of Columbia, USA Emory Vaccine Center – The Hope Clinic, Decatur, Georgia, USA National Institutes of Health – Clinical Center, National Institute of Allergy and Infectious Diseases Laboratory of Immunoregulation, Clinical Research Section, Bethesda, Maryland, USA Brigham and Women’s Hospital – Infectious Diseases, Boston, Massachusetts, USA Saint Louis University – Center for Vaccine Development, Saint Louis, Missouri, USA Vanderbilt University Medical Center – Infectious Diseases, Nashville, Tennessee, USA Baylor College of Medicine – Molecular Virology and Microbiology, Houston, Texas, USA
4	NCT05562323	Characterization of vaccine induced responses against monkeypox (MoVIHvax): an observational prospective cohort study	NA	NA	Enrollment: 100 Age: 18 years and older (adult, older adult) Sex: All	Germans Trias I Pujol Hospital, Badalona, Barcelona, Spain BCN Checkpoint, Barcelona, Spain
5	NCT05522296	Break-through infection following monkeypox vaccination	NA	Biological: monkeypox vaccine	Enrollment: 4638 Age: 18 years and older (adult, older adult) Sex: Male	Germans Trias I Pujol Hospital, Badalona, Barcelona, Spain
6	NCT03745131	Cohort study of healthcare workers receiving Imvanex®	NA	Other: blood draw	Enrollment: 120 Age: 18 years and older (adult, older adult) Sex: All	Blackpool Teaching Hospitals NHS Foundation Trust, Blackpool, Lancashire, UK Royal Liverpool and Broadgreen University Hospitals NHS Trust, Liverpool, Merseyside, UK Newcastle Hospitals NHS Foundation Trust, Newcastle Upon Tyne, Tyne and Wear, UK Royal Free London NHS Foundation Trust, London, UK Guy’s and St Thomas’ NHS Foundation Trust, London, UK
7	NCT05567939	Clinical, virological, immunological, psychosocial and epidemiological consequences of human monkeypox virus (ProMPX)	NA	Other: blood samples Other: scabs and pus samples	Enrollment: 280 Age: child, adult, older adult Sex: All	Institut Pasteur Bangui, Bangui, Central African Republic
8	NCT05654883	New York City observational study of Mpox immunity (NYC OSMI)	NA	NA	Enrollment: 300 Age: 18–100 years (adult, older adult) Sex: All	YU Langone Health, New York, New York, USA
9	NCT05627713	Clinical and biological aspects of the monkeypox disease	NA	Other: blood sample collection Other: urine sample collection	Enrollment: 330 Age: 18 years and older (adult, older adult) Sex: All	Centre Médical de l’Institut Pasteur, Paris, France
10	NCT05058898	A One Health Study of monkeypox human infection	NA	Other: blood samples Other: SCABS and pus samples	Enrollment: 280 Age: child, adult, older adult Sex: All	Institut Pasteur Bangui, Bangui, Central African Republic
11	NCT00303225	SIGA-246 to treat smallpox	NA	Drug: vaccine: SIGA-246	Enrollment: 0 (withdrawn) Age: 18–50 years (adult) Sex: All	National Institute of Allergy and Infectious Diseases (NIAID), Bethesda, Maryland, USA

NA, not available.

In conclusion, prioritization of individuals along with the production of specific Mpox vaccine is of utmost necessary and where vaccine cannot be offered due to supply, regulatory, efficacy, and safety considerations, tracing of contacts is crucial to break the transmission chain and identify those who are at risk, for containment of this multicountry outbreak of Mpox.

## Ethical approval

Not applicable.

## Consent

Not applicable.

## Sources of funding

None.

## Author contribution

All the authors substantially contributed to this work.

## Conflicts of interest disclosure

The authors declare that they have no financial conflict of interest with regard to the content of this report.

## Research registration unique identifying number (UIN)

1. Name of the registry: Not applicable.
2. Unique Identifying number or registration ID: Not applicable.
3. Hyperlink to your specific registration (must be publicly accessible and will be checked): Not applicable.

## Guarantor

Milan Gaihre.

## Data statement

Data sharing does not apply to this article as no new data were created or analyzed in this study.

## Provenance and peer review

Not commissioned, externally peer reviewed.
